# Synthetic Vesicle-Based Drug Delivery Systems for Oral Disease Therapy: Current Applications and Future Directions

**DOI:** 10.3390/jfb16010025

**Published:** 2025-01-14

**Authors:** Pengjie Huang, Weichang Li, Jiezhong Guan, Yibing Jia, Dan Wang, Yurun Chen, Niu Xiao, Songyue Ou, Yan Wang, Bo Yang

**Affiliations:** Hospital of Stomatology, Guanghua School of Stomatology, Guangdong Provincial Key Laboratory of Stomatology, Sun Yat-sen University, Guangzhou 510055, China; huangpj23@mail2.sysu.edu.cn (P.H.); liwch57@mail.sysu.edu.cn (W.L.); gjz42286214@163.com (J.G.); jiayb6@mail2.sysu.edu.cn (Y.J.); wangd236@mail2.sysu.edu.cn (D.W.); chenyr69@mail2.sysu.edu.cn (Y.C.); xiaon23@mail2.sysu.edu.cn (N.X.); ousy3@mail2.sysu.edu.cn (S.O.)

**Keywords:** vesicle, oral disease, liposome, polymersome, extracellular vesicle

## Abstract

Oral diseases such as dental caries, periodontitis, and oral cancer are prevalent and present significant challenges to global public health. Although these diseases are typically treated through procedures like dental preparation and resin filling, scaling and root planning, or surgical excision, these interventions are often not entirely effective, and postoperative drug therapy is usually required. Traditional drug treatments, however, are limited by factors such as poor drug penetration, significant side effects, and the development of drug resistance. As a result, there is a growing need for novel drug delivery systems that can enhance therapeutic efficacy, reduce side effects, and improve treatment outcomes. In recent years, drug-loaded vesicles, such as liposomes, polymersomes, and extracellular vesicles (EVs), have emerged as promising drug delivery platforms due to their high drug encapsulation efficiency, controlled release properties, and excellent biocompatibility. This review provides an in-depth examination of the characteristics, advantages, and limitations of liposomes, polymersomes, and extracellular vesicles in the context of oral disease treatment. It further explores the reasons for their advantages and limitations and discusses the specific applications, development prospects, and strategies for optimizing these vesicle-based systems for improved clinical outcomes.

## 1. Introduction

In dental treatment, there has been a growing trend of utilizing natural and synthetic macromolecules as a fundamental framework, primarily emphasizing exploring biomolecular effects at the molecular level. Broad-spectrum antibacterial antibiotics like cephalosporin have been extensively employed in the treatment of oral bacterial infections, including periodontitis, dental caries, pulpitis, and apical periodontitis [1]. Similarly, anticancer drugs such as tretinoin are utilized to treat oral leukoplakia, precancerous lesions, and oral squamous cell carcinoma (OSCC) [2]. Nevertheless, during the treatment, the therapeutic efficacy of drugs is attributed not to a single molecule but rather to their meticulously orchestrated and structurally integrated assemblies, particularly within the oral and maxillofacial region. Compared with other body parts, it presents intricate anatomical features, encompassing dental tissue, periodontal tissue, mucosa, salivary glands, and other organs or tissues [3]. Various diseases can manifest in diverse tissue types, such as dental hard tissue defects, pulpal diseases, periodontal diseases, and oral mucosa diseases. Additionally, the oral cavity represents an open and dynamic system characterized by intricate interactions. Due to difficulties in drug delivery, this dynamic nature poses challenges in achieving optimal therapeutic effects. Therefore, developing a drug delivery system is imperative for effective therapy of oral diseases [4]. Currently, medical materials used for treating oral diseases include hydrogel, which is utilized for wound healing and drug delivery in oral mucosal diseases. Cement and resin are applied to restore tooth defects caused by dental caries and pulpitis. Nickel metal wires are employed in orthodontics, and vesicles are primarily used for drug delivery and inflammation treatment. Among these materials, vesicles are the predominant choice for drug delivery in the treatment of oral diseases. Vesicles are compartments formed by a bilayer structure separating its contents from the cytoplasm or a fluid-based extracellular environment, which can be categorized into natural vesicles like extracellular vesicles and synthetic vesicles like niosomes, bilosomes, phytosomes, and polymersomes [5]. With the development of precise and accurate therapy in the oral cavity, vesicles have become a crucial component of the drug delivery system, significantly enhancing treatment efficiency and effectiveness. They have been applied in oral treatment to encapsulate various types of drugs, including small-molecule compounds [6], nucleic acids [7], proteins, CRISPR-Cas gene editing complex [8], signal molecules, and polymeric materials [9]. In the field of drug delivery, the advantages of vesicles are reflected in their broad application range and high delivery efficiency.

**Figure 1:**
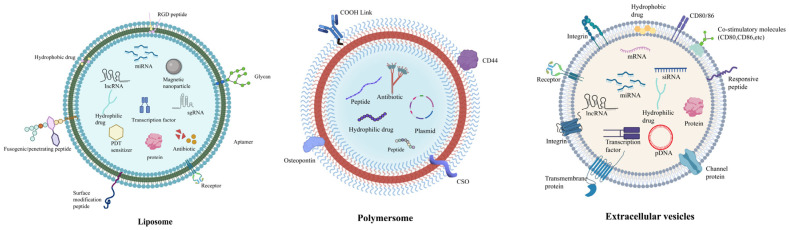
Schematic illustration of the structure of three primary vesicles.

Liposomes, polymersomes, and extracellular vesicles are the three primary types adopted in therapies for oral diseases [10]. Liposomes, initially regarded as a research model, are enclosed vesicles characterized by a bilayered phospholipid membrane structure [11]. Owing to multifunctionality and scalability, liposomes have become widely used as drug carriers in clinical practice including amphotericin B liposomes, doxorubicin liposomes, and bupivacaine liposomes. When loaded into liposomes, the drugs are embedded into a self-assembled phospholipid membrane structure, which has been used to carry various types of contents, including antimicrobial peptides [12], heavy metals [13], and small-molecule targeted antitumor drugs and proteins [14]. Polymersomes are hollow spheres formed by the self-assembly of amphiphilic block copolymers in an aqueous solution. Particularly, small-sized polymersomes (approximately 150 nm to 300 nm in diameter) can be endocytosed by cells, demonstrating excellent biocompatibility [15]. Additionally, they possess superior physical and chemical properties, such as permeability [16], stability [17], and chemical versatility [18], enabling controlled drug release. Extracellular vesicles, secreted by living cells, can be categorized into three types based on their diameter: exosomes, microvesicles, and apoptotic bodies [19]. EVs are essential bioactive molecules with significant diversity as lipid bilayer vesicles, playing a crucial role in intercellular communication. During therapy, EVs possess dual capabilities, serving as diagnostic markers and holding immense potential as intrinsic vesicles for drug delivery. They adeptly transport a variety of molecules, including oligonucleotides, proteins, and small pharmaceutical drugs, to recipient cells [20]. Research has highlighted the benefits of EVs over traditional synthetic vesicles, particularly their superior biocompatibility and reduced immunogenicity. As a result, EVs are increasingly viewed as next-generation delivery platforms. Recent studies indicate that exosomes, a specific type of EV, exhibit considerable therapeutic potential, especially in enhancing angiogenesis and facilitating wound healing. This is achieved through their role in mediating intercellular communication, cell signaling, and metabolism, owing to their cargo of mRNAs, miRNAs, and proteins [21] (Figure 1).

These three categories of vesicles have their advantages and limitations, summarized in Table 1. Liposomes, with their well-established protocols and mass production, are renowned for their easy manufacturing and tunable size [22]. Similarly, polymersomes resemble liposomes in laboratory preparation, but their scalability is limited due to the utilization of various amphiphilic polymer materials [23]. Conversely, the synthesis and isolation of extracellular vesicles pose significant challenges as they necessitate collection and isolation from living cells, rendering them highly sensitive to the environment and presenting difficulties in separation techniques [24]. Regarding treatment efficacy, original liposomes exhibit various limitations, including low selectivity and drug leakage. However, recent advancements have facilitated the modification of multifunctional liposomes with specific molecules to overcome these drawbacks, such as folate [25], cholesterol-o-nitrobenzyl-PEG [26], and magnetic Fe_3_O_4_ particles [27], which can enhance delivery efficiency and prevent leaks. With the incorporation of more biocompatible components, polymersomes and EVs have demonstrated significant potential in achieving state-of-the-art efficacy in drug encapsulation and delivery [28,29]. Liposomes have been approved by the FDA for nearly 20 clinical applications, demonstrating their ability to enhance drug efficacy and reduce toxicity. Their market is projected to generate approximately USD 7 billion in annual benefits by 2027. In contrast, polymersomes and EVs have not yet received FDA approval. While liposomes have a wide range of applications in drug delivery due to their longer history, polymersomes and EVs may offer broader prospects for development.

Various types of synthetic vesicles are fabricated from synthetic or natural polymers, often through the self-assembly of block copolymers [30]. One significant advantage of this approach is its low cost. Low-cost materials do not compromise the functionality of vesicles, and the components and design characteristics of vesicle materials offer high convenience and flexibility. This allows their structures and functions to be tailored to meet specific therapeutic needs for different diseases. Moreover, the self-assembly preparation method is considered a cost-effective and environmentally friendly technique [31]. These advantages offer greater opportunities for the appropriate functionalization of synthetic vesicles, especially in the treatment of oral diseases.

While previous reviews have extensively described the components of certain vesicles like liposomes, there remains a lack of summaries of polymersomes and EVs and their use as treatments in oral diseases. Moreover, systematic research and comparative analysis of vesicle-based carriers within the oral cavity are absent. Specifically, there has been little systematic summary of the advantages, disadvantages, and application scenarios of the three types of vesicles. Moreover, many reviews have primarily focused on a singular disease in the oral cavity rather than considering the issue from a holistic perspective. Therefore, comparing vesicle applications in oral diseases is warranted for future research and treatment advancements. In this review, we concentrate on liposomes, polymersomes, and EVs as carriers for oral diseases, aiming to unveil their mechanisms for efficient drug delivery and provide comprehensive perspectives to address specific limitations in past reviews. Our work also updates the application of these three carriers in the oral region. Finally, we analyze the existing limitations and provide guidelines for future advancements in vesicle-based drug delivery systems in the oral region.

## 2. Challenges for Treatment in the Oral Cavity

Common diseases in the oral cavity include dental defects, periodontal disease, oral mucosal disease, maxillofacial defects, oral squamous cell carcinoma (OSCC), and peri-implantitis [32]. In recent years, numerous studies have been conducted in the abovementioned fields, such as studies of their pathogenic mechanisms and drug treatment [33]. However, compared to other anatomical regions, the oral and maxillofacial region presents unique properties that pose challenges in disease treatment and restoration. The challenges include dynamic changes in the oral environment, the intricate anatomical structure, the diversity of the oral microbiome, and the high demands for the recovery of oral diseases.

The foremost challenge stems from the dynamic alteration of the oral environment and intricate anatomical structure. Situated at the entrance of the respiratory and digestive tracts, the oral cavity undergoes significant fluctuations in environmental conditions influenced by dietary habits and gas exchange, leading to variations in temperature and humidity levels [34]. After ingesting foods, the oral temperature can rise to a range of 37–40 degrees Celsius, while during respiration, it closely approximates the ambient external temperature. Additionally, the humidity within the oral cavity typically falls within the range of 30–50%, although this range is subject to alteration due to the secretion and absorption of saliva. The mechanical abrasion incurred and diluted during mastication diminishes the efficacy of localized treatment strategies due to the brief retention time within the targeted region [35]. Furthermore, the intricate anatomical structure and dynamic circulatory system frequently result in erroneous drug permeation and diminished absorption efficiency in the oral cavity. A compelling illustration can be found in the treatment of oral cancers, such as OSCC, where chemotherapy and immunotherapy medications often encounter dilution during systemic circulation [36]. The characteristics of cancers in the oral region differ from those in other parts of the body due to the unique anatomical structure, including odontogenic tumors confined to the jawbone and salivary gland tumors that invade the facial nerve. Given the distinctive environment in the oral cavity, achieving efficient drug delivery to the disease site becomes imperative for effective therapy.

The second challenge in oral treatment arises from exposure to the high diversity of the oral microbiome for the external environment, which encompasses over 700 microorganisms, including fungi and bacteria [37]. Infection by oral microbiota can manifest with pronounced clinical symptoms, chronicity infection, or asymptomatic presentation, and can disrupt the integrity of mucosal barriers [38]. The infections can be categorized into odontogenic infections, primarily from periodontitis and dental caries, and nonodontogenic infections associated with HNSCC, sexually transmitted disease, trauma, and salivary gland inflammation. With ongoing research on the relationship between oral diseases and microbiome deepening, we have identified oral diseases directly associated with the microbiome in the oral cavity. For instance, oral leukoplakia is induced by oral candidosis [39], dental caries is mainly induced by Lactobacillus and mutans streptococcus [40], pulpitis is induced by gram-negative bacteria, and periodontitis is triggered by Porphyromonas gingivalis [41]. In these oral infectious diseases, many microbial communities composed of normal and pathogenic microbiomes colonize the oral surfaces, forming complex biofilms, first employed to describe dental plaque in dental caries [42]. Microbial biofilms consist of intricate assemblies of microorganisms surrounded by bacterial and salivary-derived polymers, reducing the permeability of pharmaceutical agents [43]. Besides, the oral microbiome is implicated in the development of systemic diseases, as evidenced by the correlation between changes in oral microorganisms and diseases such as Alzheimer’s disease [44] and myocardial infarction [45], which can pose life-threatening risks. The significance and complexity of oral microorganisms present a therapeutic challenge. During treatment, antibacterial agents like cephalosporin and metronidazole are topically administered, thereby achieving augmented local concentrations in the affected region while maintaining lower concentrations within the circulatory system [46]. This localized enrichment strategy effectively diminishes systemic side effects and the emergence of bacterial resistance. Besides, existing therapies have demonstrated limited efficacy in eradicating microbial biofilms, offering only temporary relief [47]. The persistent challenge lies in the precise and continuous elimination of lesion regions while preserving the balance of microbial ecology. Hence, an efficient drug delivery system plays a crucial role in the effective antimicrobial treatment of oral inflammation. Recently, vesicle-based carriers have demonstrated effectiveness in bacterial repression, including multifunctional vancomycin-loaded sphingomyelin liposomes [48], natural EVs [49], and the capability of bacterial inhibition polymersomes [50]. Combined with specific drugs, vesicles can inhibit microorganisms and modulate the ecological balances of oral microbiota, providing a basis for future restoration.

The diverse functions of the oral and maxillofacial regions impose high demands on treatment and restoration. The oral and maxillofacial region comprises intricate anatomical structures encompassing diverse tissue and muscles, which serve vital physiological and social functions, including speaking, chewing, facial expression, and interpersonal recognition [4]. Consequently, diseases affecting this region often lead to irreversible psychological impacts and a diminished quality of life, manifested as cosmetic defects and impairment of facial expression [51]. Moreover, oral diseases afflict over 50% of the global population, presenting tremendous challenges in achieving delicate and aesthetically appealing restoration of defects and dysfunctions within the oral and maxillofacial region [52]. Nonetheless, the oral cavity harbors distinct types of stem cells and possesses an abundant blood supply, offering promising prospects for restoration [53]. To address the intricate therapeutic challenges in the oral and maxillofacial region, including precision targeting and ecological balance restoration, the integration of stimuli-responsive vesicle systems offers a promising solution. These systems, with their ability to intelligently respond to environmental changes, enhance the efficacy of drug delivery and align with the region’s functional and aesthetic restoration needs.

In the treatment of oral diseases, one of the key functions of synthetic vesicles is to regulate the release of various drugs. In recent years, the stimuli-responsive behavior of vesicles has garnered increasing attention [54]. The ability to respond to external stimuli is considered a characteristic of “smart” drug delivery systems. Under external stimuli, changes in morphology, structure, and shape may alter the physical and chemical properties of the vesicles, thereby creating favorable conditions for the “intelligent” release of drugs [55,56]. Common stimuli include pH, temperature, enzymes, oxidation/reduction, and light. In such environments, these factors can induce structural changes or degradation of the vesicles, enabling the responsive release of their payloads [57,58,59]. With advancements in biomedicine, a series of more novel stimuli-responsive mechanisms have been developed, such as electric fields, proteins, glucose molecules, ultrasound, and inflammatory microenvironments [60,61]. These types of stimuli may be applied to disease treatment in the future. Additionally, the exploration of other types of stimuli provides valuable insights for biomedical research.

Given urgent and specific requirements for functional and aesthetic restoration, developing a drug delivery system capable of large-scale production becomes imperative. Vesicle-based drug delivery systems for oral diseases and their therapeutic applications are shown in Table 2.

## 3. Dental Hard Tissue Defects

Dental diseases encompass a range of conditions, including dental caries, pulpitis, periapical diseases, and non-caries lesions affecting dental hard tissues [62]. Among these, dental caries and pulpitis are prevalent and recurrent ailments in oral healthcare settings, exhibiting a significantly high patient attendance rate at dental outpatient departments.

### 3.1. Dental Caries

Dental caries is a multifactorial disease primarily mediated by Gram-positive bacteria like Streptococcus, Lactobacillus, and Actinomyces [63]. Most caries originates from dental enamel, which contains minimal organic content, thereby primarily inducing inorganic lesions through demineralization and remineralization processes. Subsequently, pathology can infiltrate along the dentinal tubules, leading to the degradation of organic components and the demineralization of inorganic substances in dentin and cementum, ultimately resulting in the breakdown of dental hard tissues [64]. Considering the global prevalence, the prevention and management of dental caries remain formidable challenges [52], with the prevalence of untreated, permanent tooth caries in 29.4% of cases and the incidence of secondary caries in 60% of patients after undergoing polymeric restorative material treatment [65]. Currently, removal and restoration methods are widely employed for treating dental caries, but they lack efficacy in addressing deep dental caries and pose the risk of damaging nearby tissue. Certain antibacterial agents, such as doxycycline hydrochloride, metronidazole, chlorhexidine, and minocycline, are also employed in combination therapy. However, these medications are frequently associated with various adverse effects, such as tooth discoloration and taste alteration [66,67]. Therefore, the exploration of innovative and targeted treatment approaches becomes imperative.

Nisin, an antimicrobial peptide produced by the Lactococcus lactis subspecies, has been recognized for its potent inhibitory activity against Gram-positive bacteria [68]. Despite its promising antimicrobial properties, nisin faces challenges such as susceptibility to degradation and limited solubility, leading to suboptimal drug utilization efficiency [69]. Yamakami et al. validated the suitability of liposomes as carriers for nisin to enhance its stability [70]. Nisin liposomes exhibited significant efficacy in inhibiting the synthesis of insoluble glucans in mutans Streptococcus. Notably, the inhibitory concentration of nisin within liposomes was only a quarter of the concentration required for free nisin to achieve the same effect. Furthermore, the nisin liposome proved an extended inhibitory effect on biofilm biosynthesis of mutans streptococcus, maintaining this effect for 6 h. In contrast, the unencapsulated nisin gradually lost its antimicrobial activity during the identical time frame. In addition to nisin, other antibacterial drugs are incorporated into liposomes, resulting in a marked enhancement in antibacterial activity. Martínez-Gomis et al. applied liposomal lactoferrin and lactoperoxidase locally to the oral microbes and caries in rats [71]. Pinna et al. explored the preventative potential of natural antimicrobials against caries [72]. They utilized PEGylated and conventional liposomes loaded with extracts of thymus capitatus and citrus limon var. pompia, which demonstrated superior antibacterial efficacy to the standard antibiotic gentamicin. To solubilize the extracted polyphenolic compounds, glycerol was added to the liposomes as a co-solvent. These natural compounds disrupt bacterial membranes through hydrophobic interactions, enhancing permeability and causing instability [73]. Among the formulations, PEGylated liposomes, due to prolonged circulation time, displayed the most robust antimicrobial performance, with a minimum inhibitory concentration (MIC) against Streptococcus mutans below 0.078 mg/mL. The photosensitizer TBO has also been encapsulated in nanoliposomes to enhance its efficacy against Streptococcus mutans, the main cariogenic bacterium [74]. Similarly, Longo et al. conducted a clinical trial using cationic liposomes loaded with a photosensitizer called aluminum chloride phthalocyanine (AlClP) [75]. When exposed to light for 5 min, these liposomes effectively interacted with bacteria, generating reactive oxygen species (ROS) and eliminating bacteria. The study reported an average 82% reduction in bacterial load at treated sites among ten patients following photodynamic therapy. The benefits of these conventional passive liposomes are significant, as they address drug solubility and reduce adverse effects. However, their tissue-specific selectivity requires further refinement. Thus, the pursuit of more proactive transport strategies to actively target specific bacteria has been initiated.

**Figure 2:**
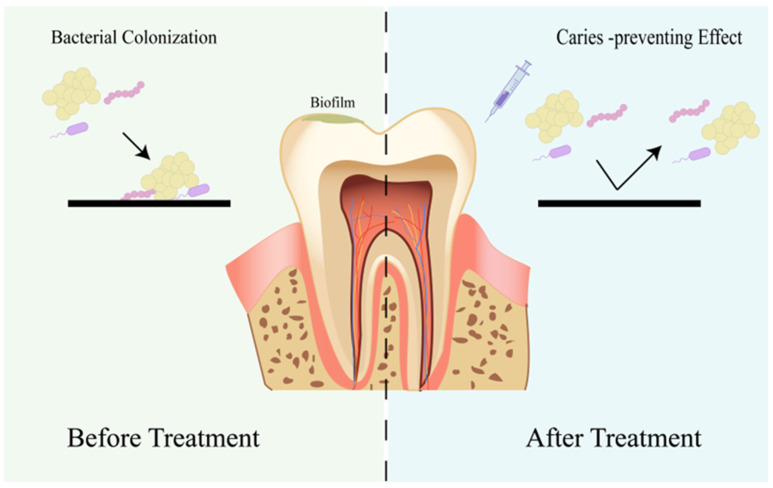
Mechanism of vesicle-based drug delivery systems in the treatment of dental caries.

Previous investigations have demonstrated the superior interactions of cationic liposomes with Streptococcus biofilms compared to conventional liposomes [76]. Microscopic observations have elucidated the heightened affinity of cationic liposomes towards bacteria and deeper penetration into biofilms, which hold potential for active delivery. Exploiting these findings, Yamakami et al. conducted further research using cationic liposomes as carriers for nisin delivery [77]. The results indicated that cationic liposomes exhibited notably higher bactericidal activity and more sustained release than anionic and neutral liposomes. Antimicrobial experiments showed significant inhibition of biofilm synthesis of glucan when incubated with cationic nisin-loaded DPPC/PS liposomes, exhibiting a 3.4-fold higher suppression than the control nisin-loaded DPPC liposomes. Tonguc-Altin et al. compared the delivery effectiveness of DOTAP cationic liposomes and Pluronic F127 copolymer hydrogel loaded with lysozyme and lactoferrin [78]. Lysozyme possesses the ability to enzymatically degrade bacterial cell walls, while lactoferrin acts to suppress the synthesis of lipopolysaccharides. When combined, the composite nanoparticle formulation exhibited a remarkable increase in inhibitory effect against Streptococcus mutans and Lactobacillus compared to lysozyme and lactoferrin alone. Zhou et al. innovatively coated doxycycline hydrochloride liposomes with quaternary ammonium chitosan (TMC), fostering interactions with negatively charged anionic constituents on bacterial surfaces and augmenting liposomal bonding to biofilms [79]. This strategic approach facilitated the active delivery of liposomes, effectively eradicating drug-resistant bacterial biofilms (Figure 2).

Similar to the principle mentioned above, another strategy for the active delivery of liposomes involves modifying components that interact with bacterial biofilm. Hu et al. harnessed DSPE-PEG-NHS, which features a carboxyl group that facilitates interactions with dental caries biofilms and supports the synthesis of peptide bonds, ultimately enabling adequate liposomal adhesion to the biofilm [80]. Thermosensitive liposomes have also been developed for delivery. Chong et al. engineered liposomes that ensure the controlled release of alkaline cargo at 36.5 °C, effectively countering the impact of the acidic environment on hydroxyapatite demineralization [81], thereby significantly mitigating erosive actions. To build on these advancements, Zhang et al. developed liposomes encapsulating histrelin-1-derived peptides, which were shown to have a more stable release and lower toxicity for the treatment of early caries [82]. Furthermore, liposome vaccines have proven effective in preventing dental caries. Childers et al. incorporated Streptococcus mutans glucosyltransferase as an antigen in liposome vaccines [83]. The result demonstrated a notable reduction in dental caries levels in animals that received oral immunization with liposomal vaccines compared to the control group treated with blank liposomes. However, passive delivery liposomes exhibit limited cellular uptake, impeding their ability to penetrate the complex barriers of humans. To address this limitation, Chen et al. developed a novel carrier utilizing anionic liposomes coated with chitosan, temporarily disrupting tight cell junctions for efficient drug delivery [84]. Through the modification, the anionic liposomes are better at stabilizing DNA and promoting enhanced cellular internalization, facilitating the targeted delivery to the nasal mucosa, thereby stimulating robust secretion of IgA and potent inhibition of Streptococcus mutans.

The application of EVs and polymersomes in dental caries therapy remains constrained. Swanson et al. developed a novel approach by integrating EVs derived from dental pulp stem cells into polymersomes composed of triblock PLGA-PEG-PLGA and diblock PEG-PLGA structures [85], thus facilitating effective EV encapsulation and sustained drug release dynamics. These composite nanoparticles were then implanted subcutaneously with a PLLA scaffold, enabling localized drug release. This strategy promoted the expression of factors related to dentinogenesis, offering promising potential for repairing damaged dentin resulting from dental caries.

### 3.2. Pulpitis

The dental pulp is a complex system comprising connective tissue, nerve tissue, and various specialized tissues responsible for preserving the tooth’s biological activity [86]. Pulpitis is inflammation or necrosis occurring in the dental pulp tissue. Odontogenic diseases, including dental caries and traumatic injuries, may result in the infiltration of deeper dentin layers, whereby stimuli are propagated through dentin tubules, reaching the dental pulp and eliciting a complex interplay of inflammatory and reparative reactions within the pulpal microenvironment, necessitating irreversible pulpectomy for symptom relief [87]. However, the one-year success rate of pulpectomy procedures is approximately 30%, and vital pulp therapy aimed at mitigating inflammation is recommended [88]. Beyond this, the integration of diverse pharmaceuticals into drug delivery systems is an emerging research focus that is currently under intense investigation. Dentinal tubules, extending from the pulp cavity to the tooth surface, display a larger diameter of approximately 2.5 μm near the pulp, tapering to 0.9~1.0 μm towards the surface [89]. This structure challenges the accession of many carriers aside from single-layered liposomes, which span a diameter from 25 nm to 1 μm, rendering them commonly employed as effective drug delivery vesicles for treating pulpitis.

The Wnt signaling pathway, a vital mediator of osteogenic differentiation that promotes cell survival, is pivotal in activating dental pulp cells to stimulate dentin regeneration [90]. Hunter et al. and Zhao et al. employed liposomes to deliver WNT3A protein to activate the Wnt signal pathway [91,92]. The outcomes revealed that WNT3A liposomes effectively stimulated dentin mineralization in dental pulp cells and upheld the viability of these cells during pulpitis. The reparative dentin demonstrated reduced dentinal tubule density compared to primary dentin, which hinders fluid flow within the tubules. Consequently, this structural alteration alleviates the impact of fluid on dental pulp nerves. Gallorini et al. used liposomes to transport ascorbic acid, β-glycerophosphate, and dexamethasone, fostering osteogenesis and modulating the microenvironment [93]. This approach accelerated the reparative dentin formation of dental pulp cells, facilitated the deposition of the mineralized matrix, and remodeled the microenvironment. Another strategy to stimulate reparative dentin formation entails regulating cellular calcium ion metabolism, a key regulator of mineralization [94]. Park et al. utilized phosphatidylserine (PS) liposomes, an anionic phospholipid that modulates cellular calcium ion metabolism via interactions with annexin proteins, to incite dental pulp cell odontogenic differentiation and mineralization [95]. The PS liposome, enriched with calcium ions, augmented in vitro biomineralization activity of dental pulp cells, serving as a potential carrier. The modulation strategies outlined above are all predicated on regulating the microenvironment of dental pulp mineralization via demineralized dentin matrix (DDM). Melling et al. employed PS liposomes to load bioactive DDM from healthy human teeth [96]. The liposomal approach exhibited a dual effect, enhancing dental pulp cell activation and reparative dentin formation by upregulating the osteogenic factor RUNX2 expression and mineralization.

In addition to dental tissue regeneration, using liposomes as drug carriers for directly loading anti-inflammatory agents is also a viable therapeutic strategy. Curcumin possesses remarkable anti-inflammatory and antimicrobial capabilities. Nevertheless, its direct application is constrained by limited water solubility and compromised photostability [97]. Sinjari et al. employed liposomes to encapsulate curcumin [98]. The curcumin-liposome was noted to elicit heightened dental pulp stem cell proliferation and attenuation of inflammatory conditions by inhibiting the NF-κB signaling pathway, thereby maintaining the homeostasis of dental pulp cells under conditions of pulpitis. Due to its cost-effectiveness and multifaceted therapy, this approach holds promise for clinical applicability. Enterococcus faecalis, a Gram-positive pathogenic bacterium, frequently contributes to persistent dental pulp infections [99]. To combat this, Ossaman et al. constructed photosensitizer 5,10,15,20-tetra(m-hydroxyphenyl) chlorin (mTHPC) liposomes for photodynamic killing [100]. Experimental findings underscored their capability against Enterococcus faecalis within root canals, with light-activatable targeting surpassing the conventional chlorhexidine (CHX) antibiotics in bacterial suppression.

The progressive harnessing of extracellular vesicles, secreted by dental pulp stem cells, underscores their therapeutic potential. Gómez-Ferrer et al. introduced HIF-1α human dental pulp MSCs with lentiviral vectors to express HIF-1α in EVs and activate immunosuppressive molecules [101]. These engineered EVs, rich in HIF-1α and immunosuppressive molecules, were highly secreted from MSCs, exerting anti-inflammatory effects in a murine model and offering a more efficient delivery system. In another application of engineered EVs from dental pulp cells, Yang et al. downregulated nuclear factor I/C (NFIC), a pivotal transcription factor in tooth root formation, within EVs [102]. Through the same method, NFIC was encapsulated into EVs of DPCs. Encapsulating NFIC into DPC-derived EVs upregulated differentiation capability, evident in increased odontoblastic differentiation and collagen production in the extracellular matrix post-NFIC-EV treatment. Given the distinctive attributes of the dental pulp chamber, the prioritization of drug-loaded vesicles’ targeting specificity is attenuated. Stability and alignment with the dimensions of dentinal tubules assume greater importance. In this context, the adoption of diminutive-sized polymeric and extracellular vesicles emerges as a promising choice, leveraging their inherent antimicrobial and osteogenic properties. Looking ahead, the integration of DPSCs with engineered EVs holds great promise in the realm of dental regenerative medicine. The synergistic application of these two modalities could revolutionize treatments for dental conditions, particularly in tooth root and pulp regeneration.

## 4. Treatment for Oral Candidiasis

Amidst the many fungal organisms inhabiting the human oral mucosa, Candida species predominate and are detectable in approximately 60% of healthy individuals’ oral cavities [103]. While generally commensal, Candida can transition to a pathogenic state under compromised immune states or oral microbiota imbalance [104]. This transformative behavior designates C. albicans as the primary species accountable for the onset of oral candidiasis. Notably, the prevalence of oral candidiasis has displayed an ascending trajectory in recent times, primarily attributed to the escalating incidence of AIDS and pharmaceutical-induced immunosuppression [105]. Additionally, it has emerged as a predisposing factor for oral squamous cell carcinoma and oral leukoplakia [106,107]. Clinical manifestations of oral candidiasis include xerostomia, mucosal adhesion sensation, oral mucosal burning, pain, and hypogeusia. The treatment of oral candidiasis typically revolves around the administration of antifungal agents. However, in stark contrast to the abundance of antibacterial medications, the array of antifungal agents suitable for oral candidiasis treatment remains confined [108]. Amphotericin B, a commonly used antifungal, has significant limitations, including cytotoxicity and poor aqueous solubility [109].

To overcome these challenges, liposomal drug delivery systems have shown significant promise in improving efficacy and reducing adverse effects. Walsh et al. compared the therapeutic efficacy and adverse effects of liposomal amphotericin B and voriconazole in treating oral candidiasis among 849 patients (~30% cure rate for severe infections) [110]. However, liposomal amphotericin B was associated with an elevated risk of renal toxicity and an increased susceptibility to hypokalemia. To mitigate these concerns, K. Wasan et al. developed liposome formulations utilizing Peceol/DSPE-PEGylation and electrically neutral lipids composed of triglycerides and fatty acids [111]. PEG-modified liposomes demonstrated superior dissolution stability and antifungal efficacy compared to neutral lipid-based formulations. Among these, liposomes incorporating PEG2000 and Peceol demonstrated superior capacity in maintaining drug stability in simulated gastric fluid. This was attributed to their longer hydrophilic chains, which interacted with the external water interface, while Peceol stabilized the encapsulated amphotericin B. Notably, this PEG-complexed liposomal formulation exhibited no significant renal toxicity in rats, as evidenced by the absence of elevated creatinine levels. In the 2012 ESCMID treatment guidelines, liposomal amphotericin B was recommended for treating candidiasis due to its recognized efficacy and safety profile in the context of pharmaceutical intervention [112].

Polymersomes have also been harnessed as carriers for amphotericin B, exhibiting minimal cytotoxicity and controlled drug release. Jain et al. conducted a systematic in vitro evaluation of (PEG)3-PLA polymersomes, focusing on the optimization of synthesis conditions [113]. Using the solvent injection method, they achieved stable vesicles. Drug release studies revealed a biphasic pattern, with an initial rapid release within 24 h, followed by sustained release, significantly outperforming conventional formulations such as Fungizone regarding hemolysis reduction and stability. Then, they evaluated the in vivo efficacy and safety of amphotericin-B-loaded polymersomes (PAMBO) in a murine model [114]. Their findings highlighted that PAMBO exhibited favorable pharmacokinetics with sustained drug levels in plasma and target organs, significantly reduced toxicity, and superior fungal clearance compared to Fungizone. Another widespread drug used in antifungal treatment is atorvastatin, known primarily for its cholesterol-lowering properties. Atorvastatin exhibits promising antifungal effects by inhibiting ergosterol synthesis, a vital component of fungal cell membranes [115]. Unlike conventional antifungal agents, including nystatin and fluconazole, atorvastatin exhibits efficacy against resistant Candida strains. Specifically, atorvastatin’s antifungal activity was comparable to or lower than the therapeutic serum levels for treating hyperlipidemia, emphasizing its practical applicability in clinical settings. To enhance atorvastatin’s delivery and efficacy, Nour et al. developed a drug delivery system combining ATV–propylene glycol liposomes with 3D-printed bioactive hydrogel films containing polyvinyl alcohol for adhesion to oral mucosa and localized release [103]. Propylene glycol liposomes enhanced penetration through cellular membranes, enabling deeper tissue delivery. These liposomes provided sustained drug release and superior antifungal activity compared to atorvastatin alone. Moreover, in conjunction with the 3D printing materials, they can mitigate oral mucosal inflammation, presenting a dual therapeutic strategy for antifungal and anti-inflammatory effects. While promising, the clinical application of atorvastatin for fungal infections requires further studies to fully explore its efficacy across diverse patient populations and fungal species.

During oral candidiasis infections, the biofilm environment typically comprises fungi and intricate interactions between fungi and bacteria [116]. Alizarin, a broad-spectrum antimicrobial agent, demonstrates dual efficacy against fungi and bacteria. In a similar approach, Raj et al. utilized chitosan gel and gum arabic (GA) as carriers to enhance the solubility of alizarin-loaded liposomes, constructed with soy lecithin and cholesterol to modulate the fluidity [117]. With enhanced alizarin’s solubility, biofilm penetration, and sustained release, the formulation reduced biofilm formation and hyphae growth at much lower concentrations. To enhance therapeutic efficacy, researchers have explored liposomes that can be used for combined drug delivery. Bandara et al. co-encapsulated N-3-oxo-dodecanoyl-L-homoserine lactone (C12AHL) and fluconazole within vesicles, enabling co-delivery [118]. The liposomal formulation loading two agents exhibited a reduction of 60% in biofilm metabolic activity compared to untreated control samples. In contrast, liposomes containing only fluconazole and unconstrained fluconazole, administered at equivalent concentrations, displayed a mere reduction of 36% and 2%.

Both oral mucosal cell-derived extracellular vesicles and Candida albicans endogenous extracellular vesicles have been shown to influence the growth and development of drug resistance in oral candidiasis [119,120]. Zarnowski et al. utilized ESCRT techniques to regulate the encapsulation of proteins into Candida albicans’ EVs to investigate their inhibitory effects on Candida albicans [121]. The absence of ECM33, ERV25, PRA1, or ZRT2 proteins in extracellular vesicles reduced drug resistance in Candida albicans due to their role in biofilm formation. Multiplying cellular vesicles to modify their inherent functionalities presents an intriguing avenue for targeted drug delivery. Vesicle-based drug delivery systems, such as polymersomes and EVs, offer novel strategies for treating oral candidiasis. Future advancements may involve the synthesis of polymersomes with enhanced permeability, adhesion, and stimuli-responsiveness, facilitating improved penetration into fungal biofilms while minimizing systemic toxicity. With their inherent biocompatibility, host-derived EVs provide a low-immunogenic platform for antifungal delivery and may be further optimized through surface modifications targeting fungal cells. Additionally, fungal-derived EVs could be investigated as innovative therapeutic carriers for antifungal drug delivery. These approaches can potentially overcome the challenges of biofilm-associated drug resistance and ineffective localized drug release, offering precision therapies for fungal infections.

## 5. Periodontitis

**Figure 3:**
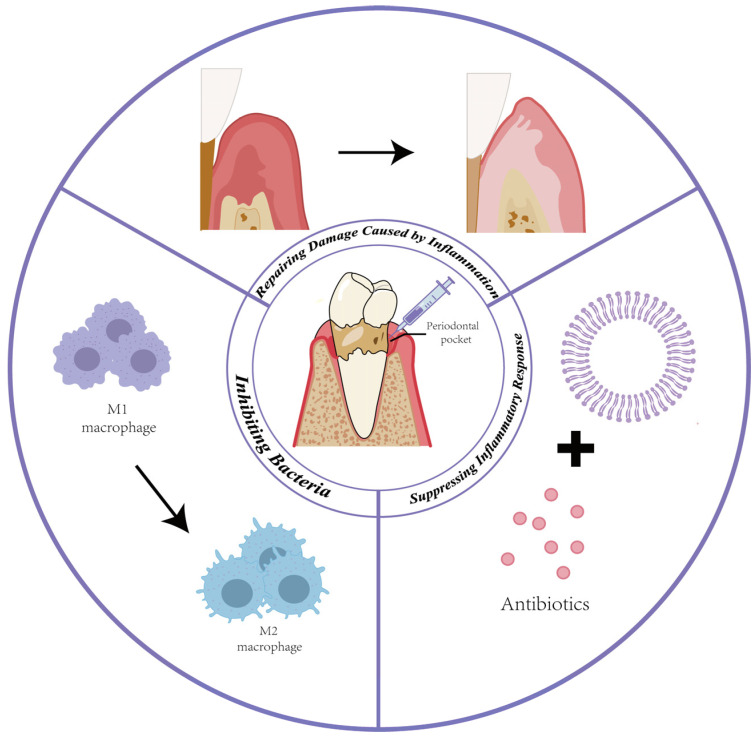
Applications of biomembrane-based nanostructures in the treatment of periodontitis.

Periodontitis, primarily initiated by bacterial infections and located in the gum, represents a high-prevalence condition, affecting approximately 50% of the global population [122]. Its pathogenesis primarily arises from the disruption of microbiome balance, leading to a shift from predominantly gram-positive to gram-negative species of bacteria [123]. With the accumulation of excessive dental plaque and dental calculus, pathogenic microorganisms activate the adaptive immune system, triggering detrimental reactions [124]. These immune responses include the differentiation of macrophages towards the proinflammatory M1 subtype, differentiating helper T cells into Th17 cells, and the secretion of proinflammatory cytokines such as IL-17, IL-6, and TNF-α by immunocytes [125]. Significantly, periodontitis extends beyond the degeneration of periodontal tissues and permanent tooth loss; it also holds the potential to contribute to systemic diseases like colitis [126], rheumatoid arthritis [127], and Alzheimer’s disease [128]. Consequently, addressing periodontitis presents three primary challenges: inhibiting bacterial growth, mitigating the inflammatory response, and facilitating the reconstruction of periodontal tissues. Conventional treatment methods for periodontitis involve mechanical scaling and root planning, together with the use of antibiotics [129]. These methods have drawbacks, such as low accumulation at the target site, inaccurate targeting, and mechanical damage to healthy tissue. To overcome this limitation, more effective vesicles for transporting therapeutic agents need to be developed [130]. Vesicle-based carriers provide a potential therapeutic system and repairment for periodontitis (Figure 3). Based on their targeted capability, liposomes, polymersomes, and EVs have been widely used in this field.

### 5.1. Inhibiting Bacteria

The most common strategy for inhibiting bacteria is the application of antibiotics, such as ciprofloxacin [131], betamethasone [132] and doxycycline [133]. With the cytotoxicity and low absorption efficiency of antibiotics, early studies used conventional liposomes to overcome the disadvantages. Early studies addressed these issues by encapsulating antibiotics within liposomes, enhancing their stability and delivery. Yet, conventional liposomes showed incomplete bacterial eradication and insufficient drug loading [134], highlighting the need for novel vesicle-based carriers.

One targeting strategy without external stimulation was to utilize the negative charge on the surface of bacteria due to extracellular proteinase secreted from bacteria. Fang et al. evaluated the antibacterial property and cytocompatibility of pH-activated Chitosan-liposome encapsulating doxycycline [135]. As a pH-responding component, N, N, N-Trimethyl chitosan with a permanent positive charge was introduced to coat liposome vesicles. Due to the low pH of the pathological condition, the pH-activated liposomes accumulated on the microbial membrane surface and penetrated the biofilm, which enhanced the antimicrobial effect of doxycycline and reduced bacterial plaque in the tooth root. Conventional single corona polymersomes were usually insufficient to inhibit bacteria owing to lacking the multifunctionalities for coping with biofilms’ intricate structure. Yuejing et al. designed dual-corona polymersomes using multifunctional copolymers, achieving bacteriostatic effects with reduced antibiotic dosages and milder inflammatory responses [136,137]. In addition, Kornchanok et al. introduced polymersomes with pH-sensitive poly[2-(diisopropylamine) ethyl methacrylate] (PDPA) components and biocompatible poly[2-(methacryloyloxy) ethyl phosphorylcholine] (PMPC) [138]. These vesicles delivered antibiotics like metronidazole and doxycycline directly to oral keratinocytes, solving the problem that antibiotics seldom penetrated the cell membrane and therefore were ineffective at eliminating intracellular microbes. Zahra Saeidi et al. combined thermosensitive gels with nanovesicles for the temperature-controlled release of encapsulated drugs. He prepared thermosensitive and mucoadhesive gels containing solid lipid nanoparticles loaded with fluconazole and niosomes loaded with clindamycin, which have more stability release and have great potential against periodontal pathogens [139].

Although engineered EVs have been widely used in treating various diseases [21,140], only a few have been applied to carry antibiotics to inhibit bacteria in oral inflammation. Natural EVs, however, have excellent specific antimicrobial properties, such as EVs derived from oral mucosal epithelial cells inhibiting candida albicans [141] and bacterial outer-membrane vesicles inhibiting gram-negative bacteria [142] primarily causing oral inflammation. Additionally, host-derived EVs can modulate the microenvironment via microRNA (miRNA) interactions, offering the potential for periodontitis treatment [143]. For instance, engineered EVs have been explored for miRNA delivery to regulate transcription and combat microbial dysbiosis [144].

### 5.2. Suppressing Inflammatory Response

Anti-inflammatory drugs terminate the progression of periodontal disease by regulating the body’s immune inflammatory response [145]. Due to the complex inflammatory regulation, common drugs applied to periodontitis, such as hydrogen peroxide [146], minocycline hydrochloride [147], and immunosuppressants, partly relieved symptoms without controlling disease progression and serious side effects [148,149]. Topical agents such as compounded iodine glycerol [150] and minocycline hydrochloride ointment [151] are commonly used to suppress the inflammatory response by slow release at specific tooth sites. However, studies showed that the release rate of minocycline hydrochloride ointment was unstable, with 80% to 90% of the drug usually released within 2 to 3 days of placement in the periodontal pocket, resulting in large fluctuations in the concentration of the drug, which was not conducive to the control of periodontal infection [151]. The strong acidity of the drug might also prevent the repair and regeneration of periodontal tissue. Therefore, the novel drug delivery system focused on regulating the oral inflammatory microenvironment, especially for macrophages, by encapsulating drugs to reduce cytotoxicity and maintain drug concentrations.

M1 macrophages mainly produce oxygen-derived free radicals (ODFR), effectively killing bacteria, but can also induce excessive inflammation, promote osteoclast differentiation, and cause tissue damage [152]. Petelin et al. employed liposomes to deliver oxygen-free radical scavengers with the ability to eradicate ODFR including superoxide dismutase (SOD) or catalase (CAT) towards the periodontal immune microenvironment [153,154]. The result demonstrated that the liposomes with SOD effectively removed the excessive ODFR and suppressed inflammation. Another application for therapy in the immune microenvironment that eliminates ROS was concentrated on remodeling macrophages from M1 proinflammatory to M2 phenotype. Junyu et al. designed liposomes loading resveratrol (RSV), which repolarized macrophages to ameliorate inflammatory reactions by activating p-STAT3 and inhibiting the p-STAT1 signal pathway [155,156]. This Lipo-RSV exhibited lower cytotoxicity and superior macrophage repolarization effect than free RSV. In addition, through the elimination of ROS in macrophages, the immune microenvironment was remodeled by Lipo-RSV, evidenced by the descent of proinflammatory cytokines.

For pathways related to inflammatory responses, nuclear factor κ-B (NF-κB) is the primary regulator to induce inflammation reactions, such as promoting monocytes and macrophages differentiation to osteoclasts and secretion of proinflammatory cytokines TNF-α [157]. Ozkan et al. developed cinnamic acid-loaded liposomes, which inhibited NF-κB activity, significantly reducing inflammatory cell proportions [158]. Targeting the inflammation factor secreted by macrophages was also a treatment option [159]. Liu et al. showed that 2% minocycline hydrochloride liposome controlled release gel significantly inhibited the production of tumor necrosis factor α (TNF-α) induced by rat macrophages [160]. The histological observation also showed that new bone and some periodontal membrane fibers were formed due to the regression of inflammatory responses. Another application by Kawazoe et al. [161] targeting TNF-α was bovine lactoferrin (bLF) encapsulated by hydrating dietary soy phosphatidylcholine multilamellar liposomes. The reduction of TNF-α generated a considerable decrease in the proportion of osteoclasts. Another study demonstrated that curcumin extended-release liposomal gel enhanced antioxidant capacity and alleviated the inflammatory state of macrophages in periodontal tissues by reducing mediators such as TNF-α and IL-1β, thereby improving the clinical outcomes of periodontitis in diabetic patients [162]. To enhance the effectiveness of transport, Dou et al. designed a type of composite engineered EVs [163]. Anti-inflammatory drugs like microRNA-21 and curcumin were loaded in mesoporous silica nanoparticles (MSNs), which were later combined with apoptotic bodies from T cells. The constructed chimeric apoptotic bodies actively targeted macrophages in the inflammatory environment for engulfment by macrophages and stimulated macrophages’ M2 polarization through the effect of molecule drugs. Compared to conventional liposomes, engineered apoptotic bodies secreted from T cells had better dual-target ability, with T cells’ innate capacity to inflammatory areas and macrophages’ selective absorption of apoptotic bodies.

### 5.3. Repairing Damage Caused by Inflammation

Periodontitis can lead to severe periodontal tissue damage, irregular bone resorption, teeth root bifurcation lesions, and subosseous defects [164]. Currently, treatment mainly involves the surgical removal of residual calculus and the application of bioactive regenerative materials to reconstruct the physiological shape of the alveolar bone [165]. The bioactive molecules, including enzymes [166] and growth factors [167], possess specific biological effects for facilitating tissue repair. However, when applied directly or combined with only hydrogel, the molecules have a low conversion rate due to quick dilution and low selectivity [168]. When engulfed by cells, clodronate can be transformed into a nonhydrolyzable analog of ATP, leading to apoptosis [169]. Michalski et al. formulated clodronate-loaded liposomes to target macrophages in periodontal pockets, reducing osteoclast-derived bone resorption [170]. This intervention created a favorable microenvironment for bone repair, although the effects varied by skeletal location. Compared to passive release, controlled release based on stimulus-response characteristics exhibits precise targeting. Sugano et al. designed ultrasound-responsive liposomes delivering plasmid DNA to gingival tissues, achieving effective periodontal treatment at an ultrasound intensity of 2 W/cm^2^, as shown by increased luciferase activity [171]. Similarly, Zinger et al. constructed collagenase nanoparticles activated by calcium. Collagenase is a matrix metalloproteinase that regulates collagen degradation in the extracellular matrix, with a short half-life [172,173]. Through the local control release of liposomes, the activation of collagenase was significantly prolonged, improving the remodeling process of the periodontal membrane fiber. Another study also exploited liposome’s controlled properties to prolong the retention of growth factors in that excessive decomposition. Ferreira et al. designed liposomes composed of dipalmitoyl phosphatidylcholine and phosphatidylcholine to carry TGF-β1 and BMP-4 [174]. The liposomes’ sustained release protected growth factors from protease hydrolysis and excessive absorption by tissue, enhancing its effects on stem cell adhesion and osteogenic differentiation. Compared to direct injection, the growth factors carried by liposomes avoided the side effects of bacterial infection caused by the massive release of growth factors, which enabled the restoration after oral inflammation.

### 5.4. Multi-Functional Vesicles for Combined Therapy in Periodontitis

As periodontitis is a complex disease involving multiple complications, such as bacterial infections, inflammation, and tissue damage, single drugs often fail to comprehensively control its progression from multiple aspects. Owing to the potential for modification, vesicles provide more possibilities for delivery. Sashini et al. developed composite liposomes to deliver ciprofloxacin, a water-soluble antibiotic, and betamethasone, a hydrophobic anti-inflammatory corticosteroid [175], solving the problem that hydrophobic medications were difficult to integrate into conventional liposomes. 1,2-dipalmitoyl-sn-glycerol-3-phosphoethanolamine (PE) and 1,2-dipalmitoyl-sn-glycero-3-phospho (PG) provided hydroxyl and amino groups on the surface to combine with the hydrophilic drug, and the hydrophobic betamethasone was encapsulated inside the liposomes. In the study, compared with carrying a single drug, the co-delivery vesicles showed better antimicrobial and anti-inflammatory properties in the treatment, suggesting a new strategy for co-delivery of drugs in multifunctional vesicles. Zhou et al. constructed antibacterial polymersomes to carry the bone growth factor BMP2 [176]. By contrast with antibiotics, the polymersomes exhibited better antibacterial activity and boosted the restoration of bone, with a great therapeutic effect in periodontal bacterial environments. Deniz Atila et al. developed a liposome-loaded hydrogel formulation with a controlled release of curcumin and α-tocopherol. The liposomes increased the stability of hydrophobic drugs, allowing curcumin and α-tocopherol to exert synergistic antimicrobial and anti-inflammatory effects. The formulation also promoted biomineralization, providing a multifunctional approach to treating periodontitis [177].

## 6. Treatment of Tumor in the Oral Region

Oral squamous cell carcinoma (OSCC) develops in the oral mucosal epithelium, accounting for approximately 90% of oral malignancies [178]. It has a high incidence and poor prognosis. About 300,000 new cases are diagnosed globally each year, and the 5-year survival rate for patients with oral squamous carcinoma is no more than 60% [179]. Most chemotherapeutic drugs have cytotoxicity, such as alkylating agents causing DNA damage and antimetabolite drugs interrupting normal cellular metabolism [180]. Conventional therapeutic methods, such as intravenous injection, make it difficult to concentrate the medications on tumor tissue, resulting in toxicity from the residual drug in the circulatory system spreading throughout the body. Long-term systemic medication can cause systemic toxicity problems, such as hepatic insufficiency and renal failure [181]. Conventional chemotherapy and radiotherapy for tumors have significant side effects on patients, leading to a reduced prognosis and seriously affecting the quality of life [182]. Innovatively, to reduce this side effect, vesicle carrier transport is a hotspot of application. To date, liposomes doxorubicin hydrochloride and paclitaxel are available and widely used in oncology patients, accounting for approximately half of China’s liposome market share by 2022 [183]. Researchers are now developing treatment strategies to combine PD-1/PD-L1 inhibitors with nanotechnology to enhance sensitivity, safety, efficacy, and personalization [184]. Nano-formulations of PD-1/PD-L1 inhibitors could soon have a vast scope in terms of patents and clinical applications. Currently, the vesicle-based drug delivery system has been developed to carry various medications, including alkylating agents, the classic anticancer drugs doxorubicin and vancomycin, and cell-penetrating peptides, exhibiting the concentration of drugs at the tumor and low cytotoxicity to normal tissue [185].

### 6.1. Elevating In Situ Tumor Delivery

**Figure 4:**
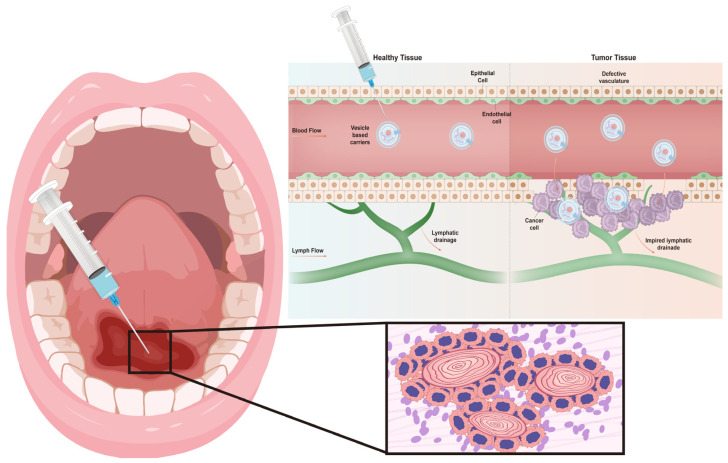
Vesicle-based drug delivery systems for targeting and therapy in OSCC.

Most vesicle-based clinically available applications were delivered via liposomes, expected to benefit patients by increasing medication solubility and managing the side effects. However, conventional liposomes, mainly through passive transportation to tumor tissue, failed to meet the requirements of clinical therapy [186,187]. Passive transportation through continuous circulation promotes targeting intratumoral delivery by tumor vascular leakage, but the detailed pharmacokinetics remain unclear. To overcome these defects, researchers focused on improving the targeting of vesicle carriers for specific tumor effects (Figure 4). For active targeting, it was common to utilize ligands binding to specific surface receptors upregulated on cancer cell membranes. To limit off-target effects, the targeted membrane receptor, such as TLR4, PDL1, and peptide–MHC complex, should be as tumor-specific as possible [188].

Resveratrol (RSV) was proven to hinder the proliferation of tumor cells by causing apoptosis [189,190]. However, its effectiveness was constrained due to RSV’s low solubility in the tissue fluid [191]. Zheng et al. constructed a dodecapeptide YHWYGYTPQNVI (GE11)-conjugated liposome bound with polyethylene glycol, utilizing the hydrophilic of PEG to improve circulation and GE11 to enhance the targeting of EGFR-overexpressing tumors [192]. Modified RSV liposome showed a 2-fold reduction in tumor volume compared to the free RSV group. Based on the principle of receptor-ligand binding to achieve active targeting, Lo et al. employed PEG-coated liposomes modified by different peptides to carry chemotherapy drugs to induce tumor cell apoptosis [193]. The multi-modified liposomes demonstrated both PH-response and cell-penetrating characteristics, providing a co-treatment strategy for tumor-targeted delivery [194,195]. Valencia et al. proposed PLGA–PEG nanoparticles encapsulating irinotecan [196]. The novel nanoparticles could target prostate cancer cells overexpressing prostate-specific membrane antigen (PSMA) by the PSMA ligand S, S-2-(3-[5-amino-1-carboxypentyl]-ureido) pentanedioic acid on the surface. The modification results in selective endocytic uptake and controlled drug release, exhibiting marked cell killing [197]. Ligand phylogeny by exponential enrichment (SELEX) is an in vitro technique used to produce aptamers that bind their targets with high affinity and specificity [198]. Based on the hypothesis that specific delivery requires screening libraries with aptamer–nanoparticle conjugates, Mu et al. explored the aptamers of the Thioaptamers (TA) with monothiophosphate-modified single-stranded oligonucleotides [199]. Through exponential enrichment (SELEX), they constructed the aptamer-liposome to deliver the TA to target HNSCC cells and identified Desmoyokin (AHNAK), a large protein, as the aptamer of TA. Loading the doxorubicin, the TA liposomes improved the transportation of doxorubicin to the HNSCC uptake-mediating surface receptors more effectively than nonmodified liposomes.

In addition, it is an effective strategy to use physical properties to improve the tumor targeting of nanocapsules. For liposomes and polymersomes, ever-expanding applications of stimuli-responsive strategies were developed to address the problem, such as light radiation stimuli, magnetic field, and ultrasound signals combined with radiofrequency ablation to break the barrier and heat release [200,201]. Li et al. constructed magnetic nano-liposomes (MLPs), with a non-toxic core composed of iron oxide [202], encapsulating dihydroartemisinin (DHA) [203], a prospective anticancer compound. To deliver MLPs to the tumor, strong magnetic needles were inserted in subcutaneous tissue in proximity to the tumor, which exhibited a better targeting effect of DHA-MLPs using a magnet. Compared with non-target DHA-MLPs and conventional liposomes, DHA-MLPs guided by the magnetic field improved the reduction of tumor growth. Photodynamic therapy (PDT) is a promising direction for tumor treatment, in which photosensitizers (PS) can kill tumors by generating free radicals after excitation. However, phototoxicity induced by PS systemic administration limits its efficacy. Ambreen et al. used photodynamic therapy (PDT) to deliver curcumin liposomes to tumors in adjuvant treatment [204]. As a result, minimal cell death was detected when utilizing curcumin-loaded liposomes without PDT, and the tumor cell death increased significantly through inducing cell apoptosis and inhibition of tumor cell metastasis when PDT was applied. Radiofrequency (RF) ablation has been successfully used in the clinical treatment of tumors at various primary sites, and its anti-tumor efficacy has been confirmed [205]. However, a common clinical disadvantage of radiofrequency ablation is the failure to eradicate the tumor [206]. To overcome this drawback, Anuradha et al. developed radiofrequency (RF) ablation combined with 186Re-modified PEGylated doxorubicin liposomes [207], which prolonged circulation time, localization to the tumor, and expanded destruction to tumor eradication. Ultrasound responsiveness is also a property that improves tumor targeting in situ [208]. Li et al. combined irreversible electroporation (IRE), a technique that uses intense electric field pulses to kill tumor cells [209], with liposome-encapsulated NVP-BEZ235, a dual PI3K/mTOR inhibitor [210]. With increasing distance from the tumor’s center, the electric field’s strength decreased, resulting in the resealing of the cell membranes after electroporation, which helps the liposomes absorbed by the marginal tumor cells to eliminate the tumor. Ramasamy et al. employed ultrasound to actively target the STAT3 decoy oligonucleotide liposome-conjugated (LPX) microbubble [211]. Under the pressure of ultrasound, LPX could be delivered only to the site exposed to the ultrasound as microbubble volume changes induced by shear stress. Additionally, sonoporation enabled the cargo to directly reach the cytoplasm, preventing being degraded by the endocytosis process.

### 6.2. Promoting Tissue and Cell Penetration

The tumor microenvironment differs from tissue fluid and plasma, in which stromal cells overproduce extracellular components, forming thick stromal obstacles and large amounts of tumor metabolites ejected from cells [212]. Hence, the tissue and cell penetration of vesicle carriers needs to be improved.

Rhenium-186 (186Re) is a common radionuclide used in chemotherapy, remotely killing cancer cells through β-emitting radionuclides [213]. Wang et al. constructed neutral liposomes and cationic liposomes to deliver Re and tested both therapeutic effects in a squamous cell carcinoma model of nude rats [214]. The results indicated that Re carried by the neutral liposomes demonstrated better tumor suppression. Liposomes enhanced the penetration of radioactive elements and allowed them to reach the tumor’s interior. In addition, the Re-neutral liposomes could follow the transit of tumor cells by detecting radioactivity, providing a means of monitoring the tumor conditions. Further evaluation was conducted on the penetration of neutral and positively charged liposomes of three different sizes at tumor sites [215]: 100 nm, 1 μm, and 2 μm in diameter. The outcome revealed that among the six groups, the 100 nm cationic liposome exhibited the highest retention rates in tumor tissue, while fluid injection activity was better in the neutral liposome group. A possible explanation for the lower efficiency of cationic liposomes was that the low PH of TME repelled the cationic liposomes [212]. When directly injected into the tumor microenvironment, the cationic liposomes decreased flow activity due to electrostatic repulsion. In another experiment, the ability of uptake and penetration of conventional liposomes, cyclodextrin-in-liposomes (DCL), and extracellular vesicles were also tested [216]. Temoporfin (5,10,15,20-Tetrakis(3-hydroxyphenyl) chlorin, mTHPC) is a photosensitizer used in the treatment of advanced OSCC. Loaded with Temoporfin, the EVs permeated deeper in OSCC spheroids and were absorbed equal to mTHPC-DCL, demonstrating excellent infiltration in TME. After penetrating the cell membrane, a drug carried by vesicles needs to enter the cell to exert its effects more effectively. Another challenge for vesicle carriers is the limited capability to penetrate plasma membranes [187]. Vesicle carriers are taken up by cells via endocytosis [217], and the lipids are ionizable at low pH, releasing their delivered drug into the cytoplasm via endosomal escape [218,219]. Hence, vesicle carriers need effective cellular absorption and endosomal escape to deliver drugs to the cytoplasm of tumor cells. To enhance cell penetration, Y. Lukianova-Hleb et al. developed clusters of solid gold spheres that could accumulate only in cancer cells to selectively insert liposome-encapsulated doxorubicin into cancer cells through conjunction with the liposome [6]. Treated with laser illumination, the gold nanoparticles generated plasmonic nanobubbles (PNBs), inducing the explosion of endosomes and drug release to the cytoplasm after being engulfed by specific tumor cells together with liposomes. PH-sensitive polymersome has also been applied to carry doxorubicin and paclitaxel into tumor cells [220]. In the study, compared with normal oral keratinocytes, internalization kinetics exhibited that based on the high expression of class B scavenger receptors on the OSCC cell surface, polymersomes were quickly internalized by OSCC when encapsulated in endosomes. After being engulfed by cells, pH-sensitive polymersomes broke down in the endosome due to the low pH and release of the anticancer drugs in the cytoplasm.

Natural EVs express proteins like tetraspanins [221] and chemokine receptors [222], which could promote fusion with the cell membrane and release cargo straight into the cytoplasm [223]. This process prevents internalization from the endosome and protects cargo from acidity. Additionally, EVs play a communication role in tumors; hence, current research mainly concentrates on suppressing or promoting the secretion of natural EVs and detecting the tumor condition through EVs. For future tumor therapy, EVs can be developed as an excellent drug delivery system for biocompatibility. Several studies on their potential as therapeutic delivery vesicles are currently in clinical trials. One study is expected to investigate the ability of plant (grape) exosomes to prevent oral mucositis associated with chemoradiation treatment of head and neck cancer. Pain caused by oral mucositis and the level of immune biomarkers in blood are listed as outcome measures [224].

## 7. Maxillofacial Bone Restoration and Regeneration

Bone restoration and regeneration are dynamic processes that repair minor bone defects or reunite minor fracture parts [225]. However, surgery for tumors and trauma, as well as orthognathic surgery (which involves the correction of facial and jaw deformities by repositioning and realigning the jaws) and other craniogenic surgeries (such as decompressive craniectomy, which involves the removal of a portion of the skull to allow the brain to swell without being compressed by the skull), cause more serious defects in the jawbone, which necessitates repair through bone tissue engineering [226]. The demand for oral implantation gradually increases due to the high prevalence of missing teeth in older people [227]. Post-implant bone tissue reconstruction is also necessary. During this treatment, vesicle-based carriers are often employed to deliver bioactive molecule drugs such as bone morphogenetic protein-2 (BMP-2) [228], transcription factors like Wnt3a [229], and hierarchical intrafibrillarly mineralized collagen (HIMC) [230] to improve the osteogenic microenvironment. Currently, vesicle-based carriers have been used in jawbone fracture repair and bioactive scaffolds for bone restoration and regeneration.

### 7.1. Jawbone Fracture Repair

**Figure 5:**
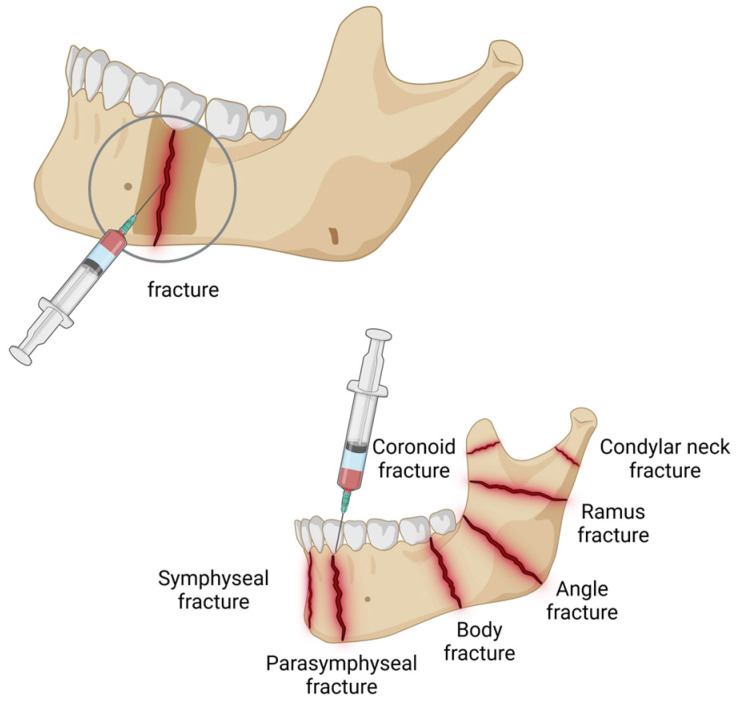
Application of vesicle-based nanostructures for fracture healing in the oral and maxillofacial region (Created in BioRender, Toronto, ON, Canada. Huang, P. (2025) https://BioRender.com/n33q877 (accessed on 6 January 2025)).

Accounting for approximately 35% of maxillofacial injuries, jawbone fractures are mainly caused by trauma, including blows, traffic injuries, and falls [231]. In addition, tumors of the maxillofacial region often result in pathological jaw fractures [232]. However, according to the anatomical location of the fractures, nearly 10% of the fractures cannot recover completely, leading to chewing disorders and social obstacles [233]. During the process of bone remodeling, the dynamic equilibrium between osteoblasts and osteoclasts mediates the growth rate of bone [234]. Therefore, vesicle-based carriers carrying drugs to improve osteogenic capacity and the microenvironment to promote fracture healing is a potential treatment option. Regulating bone homeostasis and regeneration, the canonical Wnt signaling system demonstrates outstanding potential in boosting bone density in osteoporosis patients and healing bone fractures [235,236] (Figure 5). Scarpa et al. loaded 6-bromoindirubin-3′-oxime (BIO), an activator of the Wnt signaling pathway and osteogenic differentiation [237], with polyethylene glycol–polycaprolactone (PEG-PCL) block copolymer polymersomes, which were superiorly absorbed by bone marrow stromal cells (BMSCs). The PEG-PCL polymersomes reduced the cytotoxicity of BIO and extended its circulation time to enhance targeting to BMSCs. The human multipotent stromal cells primed osteogenic differentiation with the Wnt signaling activated.

Salvianic acid A (SAA) is a potential bone-building supplement that stimulates osteogenesis and cartilage development by activating histone deacetylase 3 (HDAC3) [238,239]. Liu et al. developed bone-targeting liposomes (BTLs) using pyrophosphorylated cholesterol as a ligand to bind to hydroxyapatite in bone, effectively delivering SAA [240]. This SAA-BTL formulation enhances the uptake of SAA by osteoblasts, osteoclasts, and BMSCs, promoting maturation and differentiation in various bone tissues. As another delivery method of SAA, Zhou et al. combined SAA-BTL with collagen sponge as a scaffold framework to support the regeneration of bone cells [241]. Despite different modifications and components to attain bone tissue selectivity, most vesicle-based carriers were absorbed by the mononuclear phagocyte system (MPS) [242,243]. Ackun-Farmmer et al. constructed tartrate-resistance acid phosphatase (TRAP)- binding peptides (TBP) modified Poly(styrene-alt-maleic anhydride)-b-poly(styrene) (PSMA-b-PS) polymersomes for jaw bone fracture repair [244]. Before the TBP-polymersomes were injected, clodronate liposomes (CLO) were used to deplete the monopoly, reducing the consumption of TBP-polymersomes by MPS and enhancing bone accumulation. Vesicle transport, a mature method for transcriptional regulation and gene editing, is also an effective strategy for bone induction. Wang et al. utilized OGRU long noncoding RNAs (lncRNAs), signaling molecules with the capability of promoting osteoblast activity and matrix mineralization through the miR-320-3p/Hoxa10 axis, to induce bone formation [245].

Lin et al. generated EV–liposome hybrid vesicles to carry the CRISPR/Cas9 system to MSCs [246]. Loading big nucleic acids into EVs was challenging because of their tiny size [247], and conventional liposome delivery was constrained by efficiency [248]. Secreted from HEK293FT cells, the EVs were mixed with the plasmids and liposomes for integration. After fusion, the hybrid vesicles with a higher transfection efficacy had a larger volume to contain the large plasmid of the CRISPR/Cas9 system.

### 7.2. Combined with Bioactive Scaffolds for Bone Defects

The gold standard therapies for maxillofacial bone defects are autologous and allogenic bone transplantations [249]. However, autologous bones are constrained by the limited availability of bone sources [250]. Various materials, including metal [251], hydrogel [252], and composite materials [253], have been constructed as alternative scaffolds for bone transplantations. During the transplantation process, the bioactive bone scaffolds require drugs for inflammation elimination and bone induction [254]. When the drug is directly released in the scaffold [255], however, the concentration of the drug is uncontrollable and considerably diluted over time, such as rhBMP-2 diffused quickly from the scaffold site, with >80% dissipating within the first 24 h [256,257]. Vesicle-based carriers, as promising applications in scaffold combination therapy, could sustain drug release in the specific site of the bone scaffold.

Gazelle et al. used recombinant human bone morphogenetic protein 2 (rhBMP-2) loaded by ultrasound-triggered PEGylated liposome for the growth of synthetic implants [258]. Upon exposure to ultrasound at specific intensities, the hydrocarbon chains of liposomes were disrupted, resulting in the controlled release of their contents within the surrounding scaffold. The ultrasound-triggered liposome group exhibited increased bone formation and reestablishment compared to the control group, which was treated with the scaffold alone. Cui et al. designed a novel non-phospholipid liposome with osteoinduction characteristics [259]. The liposomes comprised 20S-hydroxycholesterol (Oxy), improving osteogenesis through activating the hedgehog signal pathway, and stearylamine (SA), a primary amine, synthesized by hydration and sonication, which reduced defect volume of calvarial bone when encapsulated in hydrogels scaffold. Similarly, Lee et al. used Oxy-SA liposome to deliver Smoothened agonist (SAG) [260], as an Hh signaling stimulator, to apatite-PLGA scaffolds in calvarial bone. In addition, the SAG liposome promoted higher-quality bone formation, as shown by the more vascularized and less pro-inflammatory macrophages, compared to autologous bone graft and BMP2. The encapsulation of extracellular vesicles in microspheres represents a promising strategy for bone restoration. Swanson et al. collected extracellular vesicles characterized as carrying proteins and microRNA with osteogenic potential from human dental pulp stem cells (hDPSCs) [261]. These extracellular vesicles were then encapsulated in microspheres composed of poly (aliphatic ester) poly(lactic-co-glycolic) acid (PLGA), a material that degrades through hydrolysis. Compared to free EVs, the EVs were encapsulated by microspheres linked to scaffolds for a longer time and were linearly released to maintain concentration. The functionalized scaffolds with EVs in microspheres stimulated bone restoration by inducing mineralization.

Inflammatory disruption induced by inserting scaffolds and previous injury in the osteogenic microenvironment inhibits bone growth and leads to bone loss [262]. To mitigate this inflammation, Wu et al. developed arginine-glycine-aspartic acid (RGD)-grafted and phosphatidylserine-containing liposomes (PSL) [263]. The PSL could be identified by PS receptors highly expressed on the macrophages, resulting in combatting inflammation through remodeling macrophages. The research exhibited that 3%-RGD-PSLs were more efficient liposomes at stimulating osteoblast proliferation and accumulation of higher-density bone components in the scaffold of bone defects. Brito Barrera et al. improved the osteogenic microenvironment for bone implant stents, including collagen type I, chondroitin sulfate, and liposomes with a positive surface charge buried inside the layer [264]. Loaded with dexamethasone, the liposomes were composed of (OO4) and dioleoylphosphatidylethanolamine (DOPE), enabling them to release their contents to surrounding cells and demonstrating high transfection efficiency. When incubated in the system, the C2C12 cells were notably differentiated into osteoblasts. Naboneeta et al. [265] utilized curcumin encapsulated in liposomes to modulate the osteoblastic microenvironment of calcium phosphate (CaP) scaffolds, which enhanced the viability and growth of osteoblast cells.

Additionally, the natural EVs demonstrate the capability of improving the microenvironment of the scaffold [266], such as increasing levels of vascular endothelial growth factor (VEGF) to increase vascularization [267] and promoting cellular-secreting extracellular matrix [268]. The dual role of natural EVs in inhibition and promotion represent a novel target for bone reconstruction. Therefore, the engineered EVs could be exploited to regulate most processes in bone regeneration.

## 8. Peri-Implant Inflammation of Dental Implants

**Figure 6:**
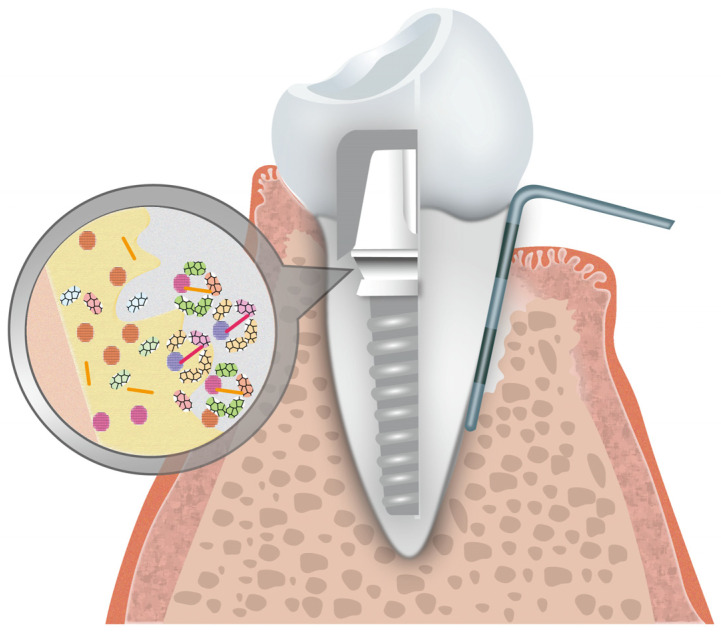
Schematic illustration of peri-implantitis.

Partly or fully edentulous patients utilize dental implants to perform their lost teeth’s function. With a frequency between 19% and 22%, peri-implantitis has become the leading cause of dental implants becoming loose and shedding [269]. Peri-implantitis, induced by the infection of bacteria, often results in inflammation and peri-implant alveolar bone loss (Figure 6) [269,270]. Yin et al. treated implants with liposome-reconstituted human WNT3A protein(L-WNT3A), which stimulated bone formation and regeneration [271]. MSCs absorbed the L-WNT3A to boost osteogenic activity around the peri-implant area to reverse the fibrous encapsulation, a sign of implant failure, around the implant. Wang et al. depleted macrophages surrounding the implant by clodronate liposome [272], a classic inflammation suppression method. After treatment, the peri-implantitis induced by Ti particle and bone absorption is reduced for a better microenvironment. De Leo et al. developed unilamellar liposome-based coatings on implant surfaces through the supported vesicular layer or modified covalently bonded vesicular layer [273]. Due to the components of anionic lipid phosphatidylserine (PS) targeting MG63 cells around the implants and promoting the formation of hydroxyapatite crystals, the liposome exhibited active drug transportation and intense interaction with surrounding cells, evidenced by fluorescence microscopy uptake accession. Similarly, Xu et al. constructed multifunctional liposomes with both bacteriostatic and anti-inflammatory properties to modify the surface of implants [274]. Loaded with minocycline (Mino), an effective antibiotic, and dexamethasone (Dex), a glucocorticoid known for its capability to reduce inflammation and promote osteogenesis, the liposome bonded to the surface stably and slowly released for 21 days to maintain the optimum drug concentration. The modified liposomes improved osseointegration around the implant, providing a new mode for drug delivery.

## 9. Patents

Liposomes, polymersomes, and EVs have their advantages as drug delivery systems in the treatment of oral diseases. A growing number of studies have begun to explore these vesicle-based systems, with some research already resulting in patent applications specifically targeting oral disease therapies (Table 3). Among these, liposome-based applications have been particularly prominent, with several patents granted and some formulations successfully translated into clinical use for oral disease treatment. The primary reasons for this success are the mature research behind liposomes, their stable nature, and their ease of production.

## 10. Conclusions and Future Perspective

In this review, we focus on recent advances in liposomes, polymersomes, and extracellular vesicles, along with an overview of their applications in the treatment of oral diseases. Due to their excellent biocompatibility and controllable performance, synthetic vesicles hold significant value for drug delivery and controlled release. In recent years, breakthrough developments in synthetic vesicles have provided ideal opportunities for the creation of multifunctional drug delivery platforms, demonstrating their potential for clinical translation. Nevertheless, as previously mentioned, although current clinical drugs have improved the efficiency of oral disease treatments, numerous challenges remain. One of the main obstacles is the complex dynamic environment and microecosystem of the oral cavity. We believe that synthetic vesicles offer a versatile toolbox for developing intelligent drug-release systems, benefiting from the diverse characteristics of liposomes, polymersomes, and extracellular vesicles discussed in this review. Selecting optimal components, structures, and properties in synthetic vesicle systems may lead to the development of novel drug delivery platforms with desirable features, including high drug-loading capacity, low toxicity, flexibility, controllability, and maximized therapeutic efficiency.

In the functional design strategies for synthetic vesicles, assemblies based on various block copolymers represent a stable matrix. The abundant active sites in block copolymers allow easy conjugation with various functional molecules, proteins, and genes, maintaining the unique structure of vesicles while enhancing their functionality. These functional assemblies are expected to address the complex clinical demands of disease treatment, particularly in terms of size effects, bioactivity, and penetrability. Although several functional designs have been developed for synthetic vesicles, their application in oral disease treatment remains in its infancy. Understanding how such nanoscale drug delivery systems participate in various cellular processes and interact with living cells is essential for developing targeted therapeutic strategies. In earlier studies, synthetic vesicle platforms often appeared overengineered to achieve multifunctionality and novelty. Once basic application needs are met, it becomes necessary to balance functionality and preparation feasibility. As research in this field progresses, clinical need-oriented approaches may become increasingly prioritized.

For the practical clinical application of synthetic vesicle systems in oral disease treatment, reliable and reproducible manufacturing techniques and scalable generalizability must be developed. Comprehensive and extensive in vivo studies of vesicle systems, including bioadaptability, biodistribution, and acute/subacute toxicity are required to ensure biosafety and feasibility. With further preclinical and clinical research, it is anticipated that deeper insights into vesicle behavior, achieved through collaboration among experts across disciplines, will expand their evaluation and application in medical fields. Adequate in vitro and in vivo data will ultimately drive the clinical translation of synthetic vesicles.

## Figures and Tables

**Table 1:** Advantages and limitations of three vesicles.

Type	Advantages	Limitations
Liposomes	Highly scalable; standardized process for production; good biocompatibility	Not suitable for bioactive modification
Polymersomes	Stable physical and chemical properties; high tissue permeability	Low membrane permeability significantly hinders the transport of drugs
Extracellular Vesicles	High biomimetic potential; inherently complicated modifications and highly tunable	Complex production process, potentially limited by source or extraction methods

**Table 2:** Oral diseases and their therapeutic approaches by vesicle-based drug delivery systems.

Oral Disease	Vesicle	Primary Cargos	Therapeutic Approaches
Dental caries	Liposomes, polymersomes	Antibiotics, lactoferrin	Inhibit bacterial proliferation and reduce demineralization
Pulpitis	Liposomes, EVs	Transcription factors, curcumin	Inhibit inflammation and promote osteogenesis
Oral candidiasis	Liposomes, polymersomes, EVs	Amphotericin B, fluconazole	Antifungal
Periodontitis	Liposomes, polymersomes, EVs	Antibiotics, microRNA, collagenase	Inhibit bacteria, suppress the inflammatory response, and repair bone damage
Oral squamous cell carcinoma	Liposomes, polymersomes,	Chemotherapeutic agents, radiotherapeutic agents, immunomodulators	Improve delivery efficiency, induce tumor cell apoptosis, and regulate the immune microenvironment
Maxillofacial bone restoration and regeneration	Liposomes, polymersomes, EVs	Signaling pathway modulators, nucleic acid, salvianic acid A	Promote osteogenesis and improve the microenvironment
Peri-implantitis	Liposomes	Dexamethasone, bioactive protein	Inhibit inflammation and promote osteogenesis

**Table 3:** The patent applications of liposomes, polymersomes, and extracellular vesicles.

S.No.	Author/Owner Patent	No./Countries Covered	Publication Date	Title of Invention	Polymer/Lipid Composition	Applications
1	Hua Yongmei; Du Jianzhong; Liao Yuyao; Wang Yue; Ni Keren; Zhao Yi	CN109620965	16 April 2019	The invention relates to a thermosensitive polymer vesicle and a preparation method and application and process thereof	PEO-b-P (NIPAM-stat-CMA)-b-PAA	Encapsulation of H2S, targeting and maintaining the proliferation and differentiation of PDLSCs
2	Van Dyke Thomas E. Holick Michael Kantarci Alpdogan Hasturk Hatice	US20130108688	2 May 2013	Delivery of H2 antagonists	phospholipid (PCs, PAs and PGs)	The delivery of H2 antagonists for preventing or treating periodontal disease
3	Xu Yan, Liu Xinhua, Gui Shuangying, Han Xu, Xu Hanying, Wang Tengfei, Hu Shaoguang, Huo Dongmei, Xin Baojian, Jiang Peng	CN112274638	29 January 2021	Specific anti-porphyromonas gingivalis egg yolk antibody liposome solution and preparation method thereof	lecithin, cholesterol, and tocopherol	To provide liposomes of yolk antibodies specific to Porphyromonas gingivalis for the treatment of periodontitis
4	Yang Deqin, Qiao Xin	CN117398361	16 January 2024	Resveratrol-loaded exosome-liposome mixed nanoparticles as well as preparation method and application thereof	lipid (PBA-COOH, EDC, NHS and DSPE-PEG 2K -NH 2) and exosome	Encapsulation of resveratrol, inhibiting periodontal disease through anti-inflammatory and immunomodulatory effects
5	Chausskaya Irina Yurevna (RU) Drobyshev Aleksej Yurevich (RU) Nikogosova Diana Eduardovna (RU) Kirilenko Vladimir Vladimirovich (RU)	RU0002826499	11 September 2024	Liposome-based photosensitizer with curcumin for photodynamic therapy	soya lecithin and carbopol	The effective delivery of curcuminoid for photodynamic therapy of inflammatory diseases of oral mucosa and periodontal tissues
6	Hou Jin Cui Zhongkai Yang Xiaojun Zhan Chaoning Tian Xin	CN114469863	13 May 2022	Application of sterol liposome as dental pulp and dentin drug delivery system	Single-stranded biparental small molecule and sterol molecule	as a drug delivery system for treating dental pulp diseases and dental hard tissue diseases.

Abbreviations: PEO: polyethylene; NIPAM: N-isopropylacrylamide; CMA: methyl coumarin hydroxyethyl methacrylate; PAA: polyacrylic acid; PDLSC: periodontal ligament stem cells; PC: phosphatidylcholine; PA: phosphatidic acid; PG: phosphatidylglycerol; PBA: phenylboronic Acid; EDC: 1-(3-Dimethylaminopropyl)-3-ethylcarbodiimide; NHS: N-Hydroxy succinimide; DSPE: 1,2-Distearoylphosphatidylethanolamine; PEG: polyethylene glycol.

## Data Availability

No new data were created or analyzed in this study. Data sharing is not applicable to this article.
